# Phytotoxic and Antifungal Effects of *Plantago major* and *Sambucus nigra* Bioextracts on Key Agricultural Pathogens: *Corynespora cassiicola*, *Fusarium oxysporum*, and *Penicillium oxalicum*

**DOI:** 10.3390/pathogens14020162

**Published:** 2025-02-07

**Authors:** Anayancy Lam-Gutiérrez, María Guadalupe Díaz-López, Jairo Cristóbal-Alejo, Nancy Ruíz-Lau, Paola Taydé Vázquez-Villegas, Mariana Valdespino-León, Ludwi Rodríguez-Hernández

**Affiliations:** 1 Laboratorio de Investigación, Tecnológico Nacional de México/ITS Cintalapa, Carretera Panamericana Km 995, Cintalapa de Figueroa 30400, Mexico; diazlopez66mg@gmail.com (M.G.D.-L.); ptayde@cintalapa.tecnm.mx (P.T.V.-V.); ludwi@cintalapa.tecnm.mx (L.R.-H.); 2 Laboratorio de Fitopatología, Tecnológico Nacional de México, Instituto Tecnológico de Conkal, Conkal 97345, Mexico; jairoca54@hotmail.com; 3 SECIHTI-Tecnológico Nacional de México, Tuxtla Gutiérrez Campus, Carretera Panamericana Km 1080, Tuxtla Gutiérrez 29050, Mexico; ruizla@conahcyt.mx

**Keywords:** phytotoxicity, antifungal activity, phytopathogens, fungal of crops, bioextracts

## Abstract

Sustainable agricultural practices increasingly focus on natural bioactive agents for managing phytopathogens. This study investigates the antifungal and phytotoxic properties of methanolic bioextracts derived from *Plantago major* leaves (MBPm) and *Sambucus nigra* roots (MBSn) to explore their potential applications. Bioextracts were prepared through methanolic maceration, with yields of 6.02% (*P. major*) and 6.42% (*S. nigra*). Antifungal assays evaluated inhibitory effects on *Fusarium oxysporum*, *Corynespora cassiicola*, and *Penicillium oxalicum*, while phytotoxicity assays assessed concentration-dependent impacts on *Solanum lycopersicum* seed germination. A qualitative evaluation of major polyphenolic compounds was conducted using Ultra-Performance Liquid Chromatography coupled with a Photodiode Array Detector and Electrospray Ionization Mass Spectrometry (UPLC-PDA-ESI-MS) to identify bioactive compounds known for their significant biological activity. *P. major* bioextracts demonstrated significant inhibition of *F. oxysporum* (90.06%) and *C. cassiicola* (83.19%), while *S. nigra* bioextracts achieved 89.65% and 92.16% inhibition, respectively. Both bioextracts showed minimal impact on *P. oxalicum*, with effects observed only at 50 mg/mL. Low concentrations of *S. nigra* bioextract enhanced seed germination, whereas higher doses inhibited it. Identified bioactive compounds included acteoside, isoacteoside, chlorogenic acid, and dicaffeoylquinic acid isomers. The findings highlight the potential of these bioextracts as biocontrol agents and modulators of seed germination processes, contributing to sustainable agricultural strategies. While this study was conducted under controlled laboratory conditions, these results provide a strong foundation for future evaluations in field settings to explore their broader agricultural applications.

## 1. Introduction

Plant-derived bioactive compounds have been extensively explored for their potential applications in agriculture, particularly in the management of plant diseases caused by phytopathogenic fungi [1]. The emergence of fungicide-resistant strains has necessitated the search for natural alternatives with potent antifungal properties [2]. *P. major* (llanten) and *S. nigra* (sauco) are medicinal plants with a long history of traditional use due to their antimicrobial, antioxidant, and anti-inflammatory properties [3,4,5,6], including applications in healing diabetic foot ulcers [7] and anticancer treatments [8,9]. However, their antifungal potential against phytopathogens that affect economically important crops remains underexplored. Furthermore, there are no reports on the antifungal properties or bioactive components of *S. nigra* roots.

Phytopathogens such as *C. cassiicola*, *F. oxysporum*, and *P. oxalicum* are notorious for causing significant yield losses in various crops [10,11,12]. These pathogens not only affect the quality of agricultural products but also pose challenges in their effective management due to their wide host range and adaptability. Investigating the antifungal properties of plant bioextracts offers a promising approach for developing eco-friendly and sustainable strategies to combat these plant diseases [13].

Although the potential of plant-derived bioactive compounds for sustainable agriculture is well documented, applying these findings beyond controlled laboratory environments poses significant challenges. Environmental variability, pathogen complexity, and scalability issues often hinder the direct application of laboratory-developed formulations to field settings [14]. Consequently, there is a pressing need for comprehensive studies that bridge the gap between laboratory efficacy and practical field deployment. This research endeavors to take a step in this direction by evaluating both the antifungal properties and the phytotoxic effects of bioextracts, offering a dual perspective on their potential utility and limitations.

The present study aims to evaluate the antifungal activity of methanolic bioextracts from the leaves of *P. major* (MBPm) and the roots of *S. nigra* (MBSn). The chemical composition of the bioextracts was characterized using UPLC-PDA-ESI-MS, a method specifically chosen for the identification of major polyphenolic compounds. These compounds are widely recognized for their diverse biological activities, including antioxidant, anti-inflammatory, antimicrobial, and phytotoxic properties, making them essential targets in the investigation of plant bioextracts with biotechnological potential. The significance of polyphenolic compounds in the food industry, agriculture, and health applications underscores their critical role in understanding the bioactive profile of the extracts under study. This study also examines the phytotoxic effects of the bioextracts on tomato seeds, providing insights into their potential applications as biocontrol agents.

By integrating antifungal assays, phytotoxicity evaluations, and detailed phytochemical profiling, this research seeks to contribute valuable knowledge about the bioactivity of *P. major* and *S. nigra* bioextracts. The findings could pave the way for developing natural fungicides and plant growth promoters, enhancing crop protection while minimizing the reliance on synthetic agrochemicals.

## 2. Materials and Methods

### 2.1. Collection of Plant Material and Processing and Extraction of Bioextracts via Continuous Maceration

The collection of *P. major* and *S. nigra* plants was carried out manually, considering their phenological stage during flowering. The collection took place in the municipality of Jitotol, Chiapas, Mexico, at the coordinates 17°05′22.0″ N latitude and 92°48′41.7″ W longitude. The region is characterized by pine/oak vegetation and predominantly features a temperate climate. The selected plant material was separated into its organs (root and leaf) and dried on a flat surface at room temperature until fully dried. Once dried, the samples were pulverized using a blade grinder (Capresso 506, Montvale, NJ, USA). Bioextracts from each plant fraction were obtained using continuous maceration with methanol as the solvent. The macerated samples were then filtered and concentrated to dryness using a rotary evaporator (Buchi, Flawil, Switzerland) at 65 °C to remove the methanol, following the protocol described by Lam-Gutiérrez et al. (2024) [15].

### 2.2. Fungal Species

Phytopathogenic strains were obtained from the fungal collection of the Phytopathology Laboratory, Tecnológico Nacional de México at the Instituto Tecnológico de Conkal. The fungal samples were isolated from stem and root lesions of habanero pepper plants.

#### Molecular Identification

The molecular identification of the strains was conducted as per the methods of Cruz-Cerino et al. (2020) [16], Hoil-Cocom et al. (2016) [17], and Moo-Koh et al. (2014) [18], which involved the analysis of the ITS1-5.8S-ITS2 region of ribosomal DNA (rDNA). DNA was extracted from mycelium grown in liquid potato dextrose medium after a 7-day incubation period at 30 °C. The integrity of the DNA was confirmed using 1% (*w*/*v*) agarose gel electrophoresis (SIGMA^®^-Aldrich, Darmstadt, Germany). The ITS1 and ITS4 primers (5′ CCGTAGGTGAACCTGCGG 3′ and 5′ TCCTCCGCTTATTGATATGC 3′, respectively), synthesized by INTEGRATED DNA TECHNOLOGIES (IDT, Coralville, IA, USA), were used to amplify the ITS1 and ITS2 internal regions flanking the 18S-5.8S-28S rDNA genes. The PCR mixture contained 1X PCR buffer, 3 mM MgCl_2_, 0.2 mM dNTPs, 2.5 units of Taq DNA polymerase, and 100 ng of template DNA in a final volume of 50 μL. The amplification process was carried out on a thermal cycler (TECHNE^®^-312 MN, Bibby Scientific Ltd., Staffordshire, UK) with the following conditions: initial denaturation at 95 °C for 2 min, 30 cycles of denaturation at 94 °C for 1 min, annealing at 54 °C for 30 s, and extension at 72 °C for 1 min, followed by a final extension at 72 °C for 5 min. The amplified PCR products were visualized on 2% (*w*/*v*) agarose gel (SIGMA^®^) and sequenced at Macrogen online (Seoul, Republic of Korea, available online: www.macrogen.com, accessed on [1 January 2009; 17 January 2017]). Sequences were compared with the GenBank database (available online: http://blast.ncbi.nlm.nih.gov, accessed on [22 October 2017; 19 January 2010]) using the BLAST tool. The identified strains were *F. oxysporum* (GenBank acc. MG020428), *C. cassiicola* (GenBank acc. GU066728), and *P. oxalicum*, identified based on morphological characteristics (ITC25). The strain of *P. oxalicum* was catalogued internally at the Instituto Tecnológico de Conkal for future reference and ongoing studies.

### 2.3. Antifungal Activity

#### 2.3.1. Standardization of the Inoculum

Strains that had been grown for 20 days were used, and a dense suspension was prepared by washing each Petri dish with 10 mL of sterile distilled water. Subsequent dilutions were performed, and the number of spores was counted using an ultraplan Neubauer chamber (Hausser Scientific, Horsham, PA, USA) and with the result expressed as spores/mL [13]. The concentration of spores was calculated using the following equation:

**Spores·mL^−1^** = Number of spores × 250,000 × dilution factor (1)

#### 2.3.2. Minimum Inhibitory Concentration (MIC) and Minimum Fungicidal Concentration (MFC)

To assess the minimum inhibitory concentration (MIC) of the bioextracts, a microdilution method was employed. A series of test tubes containing 9 mL of Sabouraud broth were prepared, with bioextract concentrations varying from 0.09 to 50 mg/mL. Each tube was inoculated with 1 mL of a fungal suspension, standardized to 5 × 10^4^ spores/mL. A negative control using the commercial fungicide chlorothalonil (Econil 720^®^ Agro Lucava, Celaya, México) at a concentration of 10 mg/mL was included to compare the efficacy of the bioextracts. Additionally, a positive control was included, which contained the fungal inoculum without the addition of methanolic bioextracts (MBPm or MBSn). After incubating the tubes for 48 h at 32 °C, the MIC was identified as the lowest concentration of methanolic bioextract that prevented any visible fungal growth. To determine the minimum fungicidal concentration (MFC), 1 mL samples were taken from the tubes that showed no growth and streaked onto agar plates. The MFC was defined as the lowest concentration of methanolic bioextract that completely inhibited fungal growth on these plates [13].

### 2.4. Effect of Bioextracts on Spore Germination

The impact of MBPm and MBSn on the germination of spores from *C. cassicola, F. oxysporum,* and *P. Oxalicum* was evaluated using the following method. Spores were obtained from 20-day-old cultures grown on Sabouraud agar plates (MFC) at 32 °C. To harvest the spores, the plates were inundated with 10 mL of sterile distilled water supplemented with 0.01% (*v*/*v*) Tween^®^ 80, and the spores were then carefully scraped using an L-shaped glass spreader [15]. The resulting spore suspension was quantified using a Neubauer chamber.

(2) $$Inhibition\;\%=\left(\frac{SGPC-SGEO}{SGPC}\right)\times 100$$

In the equation, SGPC represents the number of spores grown in the positive control, and SGEO refers to the number of spores cultured in the presence of bioextracts.

### 2.5. Phytotoxicity Assay

#### Germination Bioassay

The bioextracts for analysis were filtered using a 0.45 µm filter paper with a vacuum pump to ensure sterilization. Prior to the experiment, *Solanum lycopersicum* (Saladette variety) tomato seeds were obtained from a commercial supplier (Vita^®^ seed company, Cuernavaca, México, V-798, 0.6 g pack). The seeds were immersed in sterile distilled water for 24 h. In 9 cm diameter Petri dishes lined with filter paper, 15 seeds were placed, and 5 mL of the aqueous extract was added to moisten the filter paper completely. Three replicates were performed. The seeds were incubated at 25 °C in an incubator with 80% relative humidity, with the Petri dishes randomly distributed within the incubator for 6 days in the dark. The number of germinated seeds, as well as the fresh and dry weight, were determined. Different parameters were measured according to Ullah et al. (2014) [19] and Moreno et al. (2013) [20] and calculated as follows:

(3) Finalgerminationpercentage (FG%)=No. of germinated seeds/No. of incubated seeds

(4) Mean Germination Rate (MGR)=P1/T1+P2/T2+…+Pn/Tn

(5) MeanGermination TimeMGT=((x1 d1)+(x2 d2)+…+(xn dn))/Xn

(6) GerminationFrequency=Xi/Xn

In the above, P = number of germinated seeds; T = germination time; n = last day of monitoring; x1, x2, xn = seeds germinated on days 1, 2, … n; d1, d2, … dn; dn = days of incubation; Xi = seeds germinated per day of monitoring; and Xn = total number of seeds germinated on the last day of monitoring. The variability between the replicates is reflected in the values calculated using the equations.

The phytotoxicity experiments were conducted using a multi-level factorial design that included five treatment levels evaluated over six days. The treatments consisted of a positive control using water, a control using methanol, and methanolic bioextracts at concentrations of 5 mg·mL^−1^, 10 mg·mL^−1^, and 25 mg·mL^−1^. The experiment followed a multi-level factorial design with a 5 × 5 configuration, consisting of five treatments and three replicates for each treatment level, resulting in a total of seventy-five experimental units.

### 2.6. Identification of Bioactive Compounds by UPLC-PDA-ESI-MS

The identification of major polyphenolic compounds was conducted using a scientifically rigorous methodology, encompassing sample preparation, chromatographic conditions, and mass spectrometry data acquisition and analysis.

To ensure high-quality sample preparation, 20 mg of material was dissolved in 1 mL of methanol, followed by filtration with 0.2 μm membranes (PTFE Phenex) to remove particulate matter. Methanolic extracts derived from leaves, fruits, and roots were concentrated, with solvents removed using rotary evaporation for analytical consistency.

Chromatographic analyses were performed on a Waters Acquity H Class UPLC system (Waters, Milford, MA, USA) equipped with a BEH C18 column (1.7 μm, 100 × 2.1 mm ID). Under the conditions reported by Herrera-Pool et al. (2021) [21], the mobile phase consisted of water acidified with 0.1% formic acid (phase A) and acetonitrile similarly acidified (phase B). The gradient elution commenced with 100% phase A, transitioning linearly to 100% phase B by minute 20, at a flow rate of 0.150 mL/min. The PDA detector recorded absorbance spectra from 190 to 600 nm, focusing on the 290 nm and 350 nm channels for bioactive compound detection. A wavelength of 350 nm was chosen for its higher sensitivity to major polyphenolic compounds, which were the primary focus of this study. This wavelength provided the highest resolution for detecting the compounds of interest.

Mass spectrometry analysis utilized a Waters Xevo TQ-S micro instrument (Waters, Milford, MA, USA). Data were collected in the full-scan mode (20–2000 *m*/*z*) with negative ionization (ES-). Instrument parameters included a capillary voltage of 3.2 kV, cone voltage between 50–125 V, source temperature at 150 °C, and desolvation temperature at 350 °C with a gas flow of 650 L/h. Spectral data were matched to established databases and the literature to tentatively identify compounds of interest.

Data processing relied on the MassLynx V4.1 software (Waters, Milford, MA, USA) for peak integration, spectral analysis, and compound identification using database cross-referencing. Databases such as PubChem, ChemSpider, the Dictionary of Natural Products (DNP), and MassBank were explored for compound identification. Additionally, GNPS (Global Natural Products Social Molecular Networking) and METLIN provided complementary information for annotating specific spectral features. These resources were instrumental in identifying and validating the bioactive compounds in the extracts.

### 2.7. Statistical Analysis

The data were analyzed using the Statgraphic Centurion version XV software (Statpoint Technologies, Inc., Warrenton, VA, USA). All data were expressed as the mean ± standard deviation (SD). Statistically significant differences among mean values were determined by one-way ANOVA (*p* < 0.05). Pairwise comparisons were performed using Tukey’s test (*p* < 0.05). One-way ANOVA and Tukey’s tests were specifically used in the following two experiments: (1) to evaluate the effect of methanolic bioextracts on spore germination of *C. cassiicola*, *F. oxysporum*, and *P. oxalicum*; and (2) to analyze the phytotoxic effects of the bioextract treatments at different concentrations on plant germination and seedling growth.

## 3. Results

### 3.1. Identification and Extraction of Plant Material

Regarding the collection of the plant material, the identification of the plant material was carried out with the support of the Secretariat of Environment and Natural History (SeMAHN) of Chiapas, Mexico. The samples were deposited and are part of the scientific collection. The extraction of *P major* leaves (450 g) yielded 27.08 g of extract (6.02%), while *S. nigra* roots (410 g) produced 26.32 g of extract (6.42%). Both plant materials showed comparable extraction efficiencies.

### 3.2. Antifungal Activity

#### 3.2.1. Effect of Methanolic Leaf Bioextracts of *Plantago Major* (MBPm)

The strain *P. oxalicum* showed no inhibition with MBPm in the minimum inhibitory concentration (MIC) assay. However, a minimum fungicidal concentration (MFC) was observed at 50 mg/mL. Additionally, *P. oxalicum* exhibited sensitivity at concentrations as low as 0.43 mg/mL, with a reduction in sporulation percentage to 7.89% at 50 mg/mL, as shown in Table 1.

**Figure 1:**
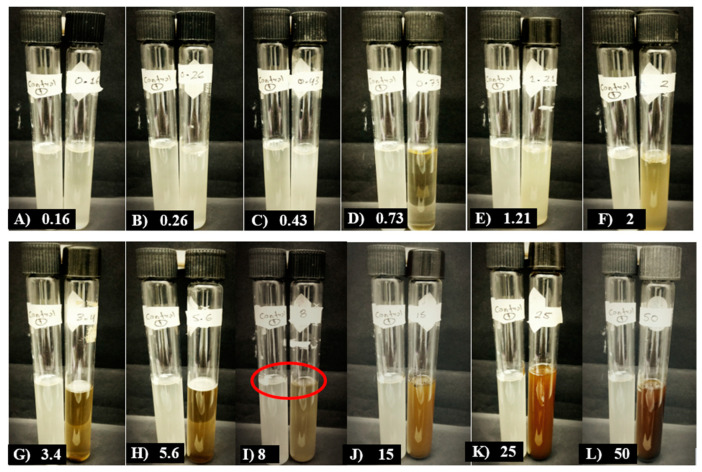
Effect of MBPm on the minimum inhibitory concentration (MIC) against *C. cassicola*. The positive control (leftmost tube) represents a sample without methanolic bioextract and contained the inoculum of the evaluated pathogen. The treatments tested included concentrations of 0.16, 0.26, 0.43, 0.73, 1.21, 2, 3.4, 5.6, 8, 15, 25, and 50 mg·mL^−1^ of MBPm. The red circles indicate the absence of visible fungal growth (spores and mycelia) and the absence of liquid turbidity, marking the MIC.

In the evaluation of the effect of MBPm on the *C. cassiicola* strain, the MIC was determined in tube I) at 8 mg·mL^−1^. Figure 1 shows that at this concentration, no fungal growth was observed compared to the positive control tube without the bioextract.

MBPm did not exhibit a value for the minimum fungicidal concentration (MFC) against *C. cassiicola*. It is important to note that starting at a concentration of 0.16 mg/mL, the percentage of sporulation was reduced to 71.64%. As the concentration of the methanolic bioextract increased up to 50 mg/mL, the inhibition percentage reached 83.19% (Table 2).

**Figure 2:**
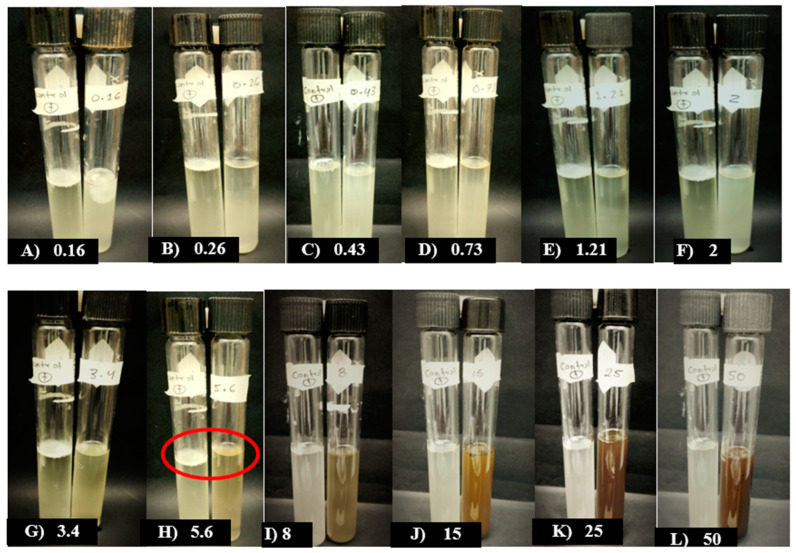
Effect of MBPm on the minimum inhibitory concentration (MIC) against *F. oxysporum*. The positive control (leftmost tube) represents a sample without methanolic bioextract and contained the inoculum of the evaluated pathogen. The treatments tested included concentrations of 0.16, 0.26, 0.43, 0.73, 1.21, 2, 3.4, 5.6, 8, 15, 25, and 50 mg·mL^−1^ of MBPm. The red circles indicate the absence of visible fungal growth (spores and mycelia) and the absence of liquid turbidity, marking the MIC.

Conversely, when testing MBPm against *F. oxysporum*, a minimum inhibitory concentration (MIC) was identified at a concentration of 5.6 mg/mL, as no fungal growth was observed when compared to the positive control, as shown in Figure 2.

MBPm did not exhibit a minimum fungicidal concentration (MFC); however, at a concentration of 0.16 mg/mL, the percentage of sporulation decreased to 62.97%. As the concentration of the extract increased to 50 mg/mL, the inhibition percentage reached 90.06%.

#### 3.2.2. Effect of Methanolic Root Bioextracts of *Sambucus Nigra* (MBSn)

MBSn did not exhibit a minimum inhibitory concentration (MIC) for the development of *P. oxalicum*, as growth was observed at all the tested concentrations compared to the negative control. Regarding the minimum fungicidal concentration (MFC), it was found at 50 mg/mL, inhibiting fungal development; however, at a concentration of 25 mg/mL, the percentage of inhibition reached 55.48%, and at 50 mg/mL, inhibition was recorded at 72.83%. While mycelial growth was observed, spore development did not occur as the concentration increased, Table 3.

**Figure 3:**
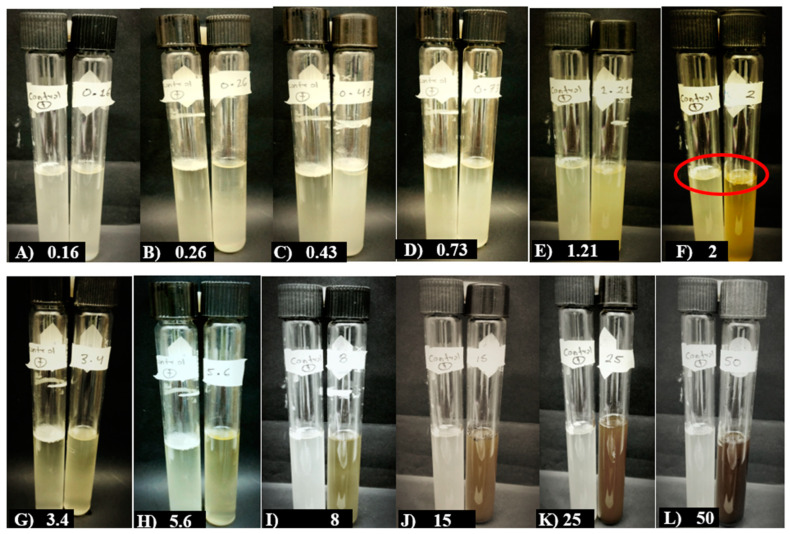
Effect of MESn on the minimum inhibitory concentration (MIC) against *C. cassiicola*. The positive control (leftmost tube) represents a sample without methanolic bioextract and contained the fungal inoculum of the evaluated pathogen. The treatments tested included concentrations of 0.16, 0.26, 0.43, 0.73, 1.21, 2, 3.4, 5.6, 8, 15, 25, and 50 mg·mL^−1^ of MBSn. The red circles indicate the absence of visible fungal growth (spores and mycelia) and the lack of liquid turbidity, marking the MIC.

MBSn demonstrated an inhibitory effect on the *C. cassiicola* strain, exhibiting a minimum inhibitory concentration (MIC) in tube F at a concentration of 2 mg/mL, as shown in Figure 3. However, no minimum fungicidal concentration (MFC) was determined. Notably, a significant reduction in the sporulation percentage was observed starting from a concentration of 0.16 mg/mL, achieving 51.72% inhibition. As the concentration of the tested extract increased, the inhibition of sporulation rose gradually, reaching up to 92.16% at a concentration of 50 mg/mL.

**Figure 4:**
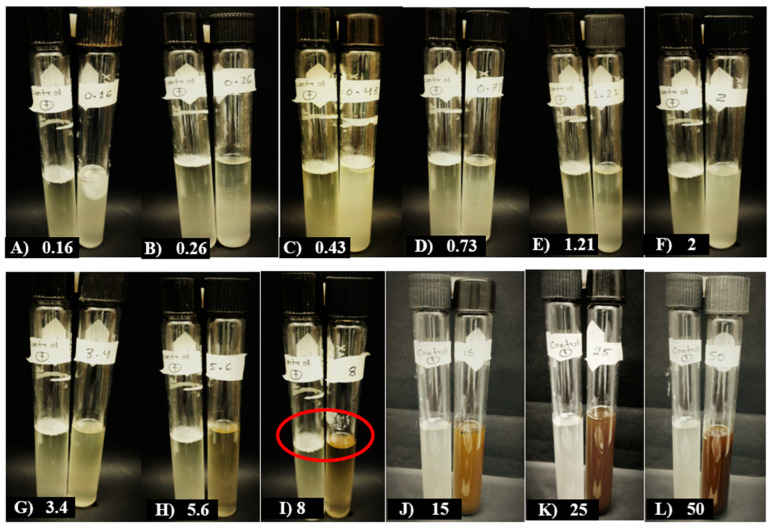
Effect of MBSn on the minimum inhibitory concentration (MIC) against *F. oxysporum*. The positive control (leftmost tube) represents a sample without methanolic bioextract and contained the inoculum of the evaluated pathogen. The treatments tested included concentrations of 0.16, 0.26, 0.43, 0.73, 1.21, 2, 3.4, 5.6, 8, 15, 25, and 50 mg·mL^−1^ of MBSn. The red circles indicate the absence of visible fungal growth (spores and mycelia) and the absence of liquid turbidity, marking the MIC.

In assessing the growth of *F. oxysporum* against various concentrations of MBSn, the minimum inhibitory concentration (MIC) was identified in tube I at a concentration of 8 mg/mL, as shown in Figure 4. At this concentration, no fungal growth was observed when compared to the positive control. A minimum fungicidal concentration (MFC) was determined at 50 mg/mL, and a concentration of 25 mg/mL reduced the sporulation percentage to 27%, while a concentration of 50 mg/mL achieved an inhibition rate of 89.65%.

### 3.3. Phytotoxicity Assay: Germination Parameters

Table 4 summarizes the effects of MBPm and MBSn on tomato seed germination parameters. For MBPm, the final germination percentage (FG%) decreased as the extract concentration increased, with values dropping from 77.77% at 5 mg/mL to 28.88% at 25 mg/mL. The mean germination rate (MGR) also declined from 3.37 at 5 mg/mL to just 2.8 at the highest concentration, indicating reduced seed vigor. The inhibition percentage rose sharply from 22.22% to 71.11% as the concentration increased, reflecting a strong dose-dependent phytotoxic effect.

In contrast, MBSn showed a distinct pattern. At a low concentration (5 mg/mL), it enhanced germination, reaching an FG% of 108.33%, surpassing the water control. The MGR was notably high at 4.46, indicating a stimulatory effect on the seed germination speed. However, at 10 mg/mL, there was a marked decrease in FG% to 61.11%, and the MGR dropped to 1.56. At 25 mg/mL, germination was completely inhibited (0% FG), showing 100% inhibition. The mean germination time (MGT) increased slightly for both bioextracts as the concentrations rose, indicating delayed germination at higher doses. For MBPm, the MGT increased from 3.57 to 6.07 days, while for MBSn, it rose from 3.46 to 4.86 days at 10 mg/mL before complete inhibition at 25 mg/mL.

**Figure 5:**
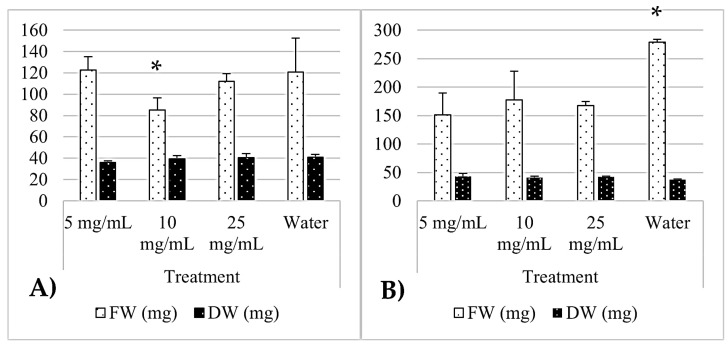
Effect of MBPm (**A**) and MBSn (**B**) on the fresh weight (FW) and dry weight (DW) of *Lycopersicon esculentum* seeds. Values are expressed as mean ± SD, with *p* < 0.05 according to Tukey’s test. Significant differences marked with an asterisk.

MBPm and MBSn showed distinct effects on the fresh and dry weights of the tomato seeds. The MBPm treatments resulted in moderate variations in both the fresh and dry weights across the concentrations, with the highest fresh weight at 5 mg/mL. In contrast, MBSn significantly increased the fresh weight at 5 and 10 mg/mL, with the maximum fresh weight observed at 10 mg/mL. The dry weights remained relatively stable across all treatments. The water control exhibited the highest fresh weight, indicating that while both bioextracts influenced seed hydration and biomass differently, MBSn had a more pronounced effect on the fresh weight at lower concentrations (Figure 5).

### 3.4. Major Polyphenolic Compounds: Identification by UPLC-PDA-ESI-MS/MS

**Figure 6:**
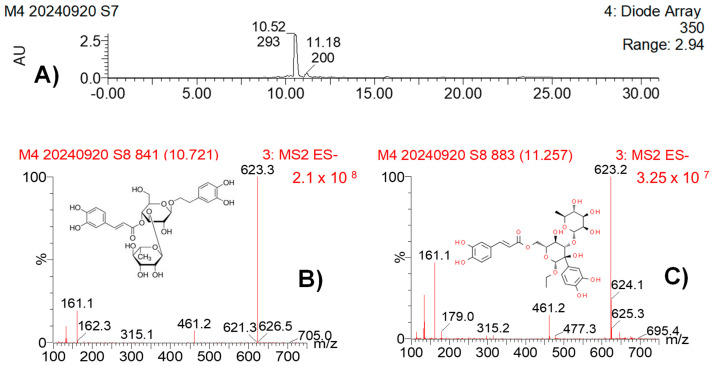
UPLC-PDA-ESI-MS analysis of MBPm: total ion current chromatogram (**A**), MS/MS spectra from peak 1 (**B**) and peak 2 (**C**).

**Figure 7:**
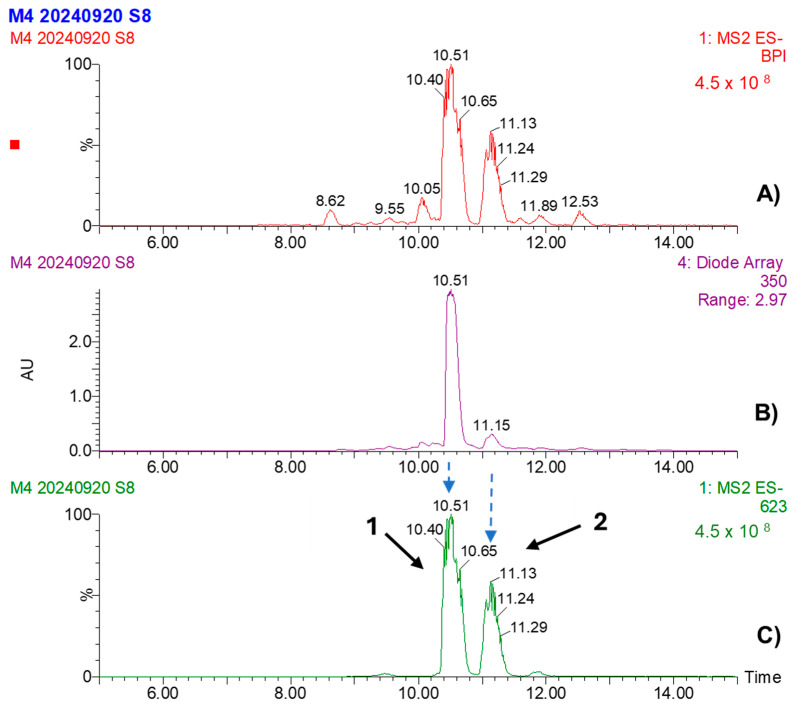
UPLC-PDA-ESI-MS analysis of MBPm: peak-intensity-based (**A**), PDA chromatogram recorded at 350 nm (**B**), and extracted ion chromatogram at *m*/*z* 623 obtained in MS/MS mode (**C**). Major polyphenolic compounds identified in peaks 1 and 2.

Table 5 presents the analysis of MBPm using UPLC-PDA-ESI-MS in the negative ion detection mode. Acteoside was detected at a retention time of 10.55 min, with λ max values at 328, 295 (shoulder peak), and 224 nm. The molecular ion [M–H]^−^ appeared at *m*/*z* 623, with characteristic fragments at *m*/*z* 461, 161, and 133. This compound (CAS: 61276-17-3) is known for its antioxidant and antimicrobial properties. Isoacteoside was identified at a retention time of 11.18 min, with maximum absorption at 327, 295 (shoulder), 218, and 197 nm. The molecular ion [M–H]^−^ was also observed at *m*/*z* 623, with similar fragments at *m*/*z* 461, 161, and 133. Isoacteoside (CAS: 61303-13-7), an isomer of acteoside, shares similar biological activities but with slight differences in its structural configuration. The identification of both compounds was based on a comparison of retention times, UV spectra, and mass spectrometry fragmentation patterns reported in the literature, confirmed by CAS references (Figure 6 and Figure 7).

**Figure 8:**
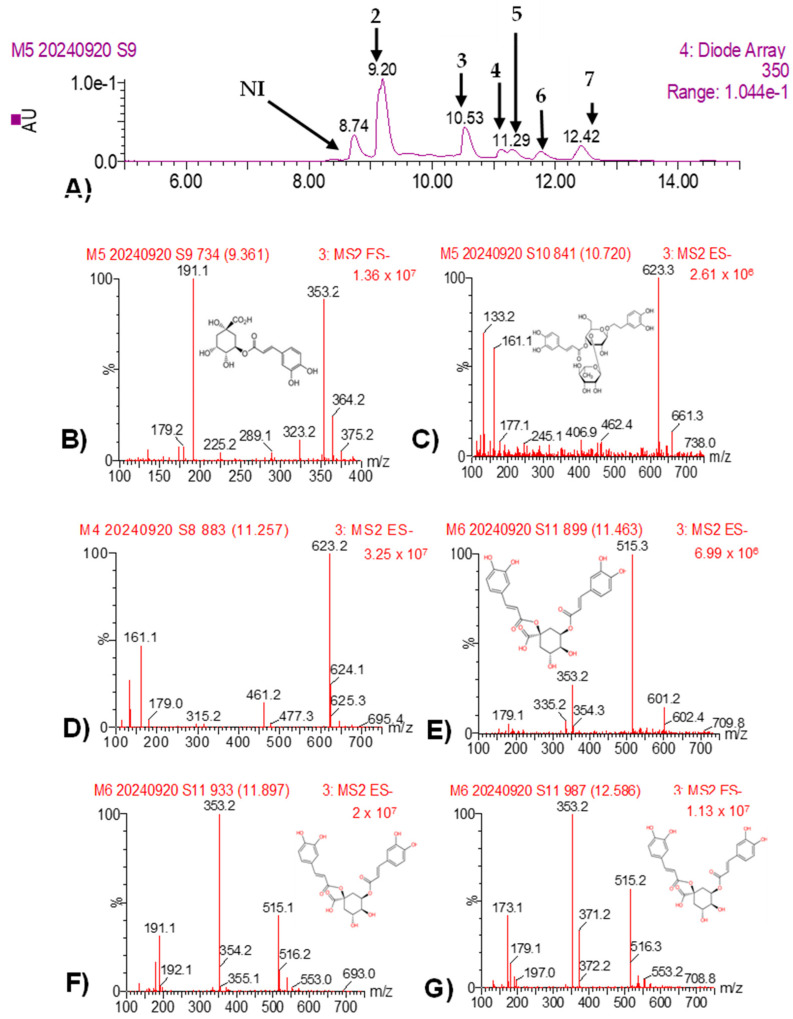
UPLC-PDA-ESI-MS analysis of MBSn: total ion current chromatogram from MBSn (**A**), MS/MS from peak 2 (**B**), peak 3 (**C**), peak 4 (**D**), peak 5 (**E**), peak 6 (**F**), and peak 7 (**G**). NI = not identified.

**Figure 9:**
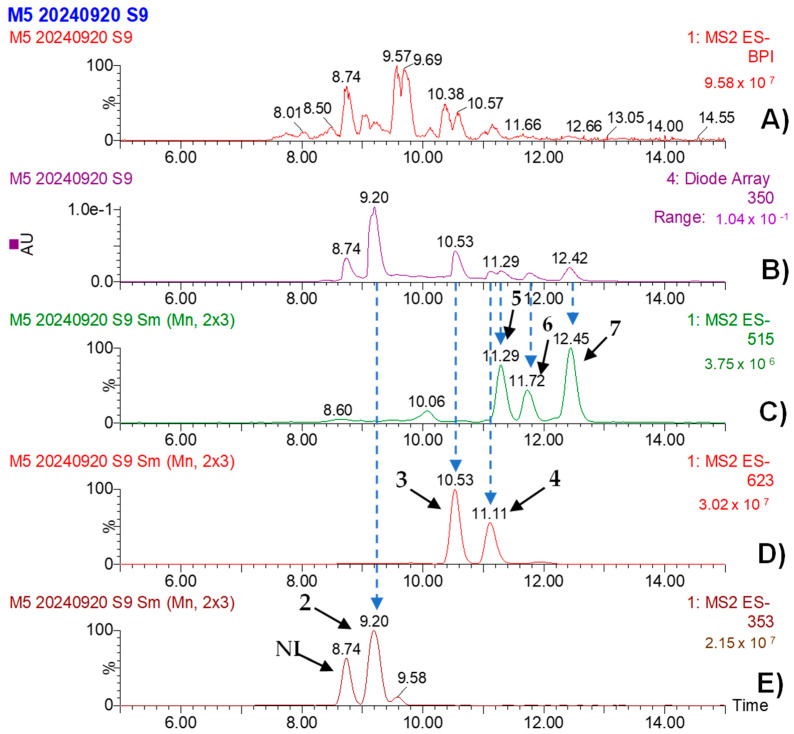
UPLC-PDA-ESI-MS analysis of MBSn: peak-intensity-based (**A**), PDA chromatogram recorded at 350 nm (**B**), extracted ion chromatogram (EIC) for *m*/*z* 515 showing peaks 5–7 (**C**), EIC for *m*/*z* 623 showing peaks 3 and 4 (**D**), and EIC for *m*/*z* 353 showing peak 2 (**E**). All EICs were obtained in MS/MS mode. Major polyphenolic compounds identified in peaks 2–7. NI **=** not identified.

The UPLC-PDA-ESI-MS analysis of MBSn revealed several bioactive compounds (Table 6), which were identified based on their retention times (Rt), UV absorption spectra, and mass spectrometry fragmentation patterns. At 9.17 min, chlorogenic acid (CAS: 202650-88-2) was identified, with a characteristic λ max at 325, 295 (shoulder), and 201 nm and a molecular ion at *m*/*z* 353, with fragment ions at *m*/*z* 191. At 10.55 min, acteoside (CAS: 61276-17-3) was identified, showing a value of λ max at 328, 295 (shoulder), and 224 nm, with a molecular ion at *m*/*z* 623 and fragments at *m*/*z* 461, 161, and 133. On the other hand, Isoacteoside was identified at 11.11 min (CAS: 61303-13-7). Three isomers of dicaffeoylquinic acid were also detected at retention times of 11.29, 11.72, and 12.42 min, with corresponding molecular ions at *m*/*z* 515 and various fragmentation patterns. These compounds were tentatively identified based on comparison with available references, including MassBank data for the dicaffeoylquinic acid isomers (Figure 8 and Figure 9).

Peak 1 was not deconvoluted due to the overlapping ionization patterns, which made it difficult to clearly distinguish individual components given the resolution of the available data. Therefore, peaks without a reliable fragmentation pattern were labeled as ’not identified’ (NI) and are presented in Table 6, as well as in Figure 8 and Figure 9, for reference.

The separation of isomers I, II, and III of dicaffeoylquinic acid (peaks 5 to 7, respectively) was performed to highlight the differences in their fragmentation patterns. These differences provide valuable insights into the subtle structural variations between the compounds (Figure 8 and Figure 9).

## 4. Discussion

The yield of plant bioextracts can vary significantly depending on factors such as the extraction method, the type of solvent used, and the specific part of the plant being processed [22]. Generally, methanolic bioextracts from leaves yield between 10% and 20% of the dry weight. In the specific case of eleuthero root, it is expected to contain no less than 3.0% of water-soluble extractives and 3.0% of alcohol-soluble extractives [23]. In this study, the values found were 6.02% for MBPm and 6.42% for MBSn. This variability underscores the importance of understanding the specific extraction dynamics of each plant species.

MBPm demonstrated varying levels of antifungal activity against different phytopathogens, including *P. oxalicum*, *C. cassiicola*, and *F. oxysporum*. While the *P. oxalicum* strain showed no inhibition at the minimum inhibitory concentration (MIC), the extract exhibited significant activity at higher concentrations, with an inhibition percentage reaching 90.06% at 50 mg/mL. The absence of a minimum fungicidal concentration (MFC) against *C. cassiicola* may suggest that the extract’s mode of action is primarily fungistatic rather than fungicidal. Furthermore, fungistatic activity can be beneficial in integrated pest management (IPM) strategies, where prolonged inhibition of fungal growth can limit the spread of diseases without exerting selective pressure that could lead to resistance.

In a study reported in [14,24], it was found that MBPm is effective in inhibiting the growth of *Streptococcus pyogenes* at doses from 250 mg/mL. Our study evaluated concentrations up to 50 mg/mL. However, the significant reduction in sporulation observed at lower concentrations is noteworthy. A fungistatic effect in plant bioextracts can be considered advantageous in the context of phytopathology. Such botanical fungicides inhibit the growth of pathogenic fungi without necessarily killing them, which can help prevent the development of resistance—a common issue associated with synthetic fungicides [25,26]. The natural origin, lower toxicity, and additional antioxidant properties of these bioextracts present potential advantages in integrated pest management and as alternatives for organic farming practices.

In contrast, MBSn showed no inhibition against *P. oxalicum*, highlighting the selective antifungal efficacy of the plant bioextracts. However, MBSn demonstrated promising inhibitory effects on *C. cassiicola* and *F. oxysporum*. The MIC for *C. cassiicola* was found to be 2 mg/mL, and the percentage of inhibition of sporulation reached 92.16% at 50 mg/mL, indicating that the root extract may be a more potent option for combating this pathogen. Similar to MBPm, MBSn showed a dose-dependent antifungal effect, with the highest inhibition recorded at 50 mg/mL for both pathogens. The presence of an MFC for *F. oxysporum* at 50 mg/mL indicates that this extract has fungicidal potential at higher concentrations, making it suitable for integrated pest management strategies. Although there are no reports on the antifungal use of MBSn specifically, studies on *S. nigra* flower bioextracts have been documented. For instance, *S. nigra* flower extract demonstrated 80% growth inhibition against *Fusarium poae* and less than 20% inhibition against *F. oxysporum* at a 10% concentration [27]. This variability suggests that the antifungal efficacy of *S. nigra* can differ depending on the fungal species and the plant organ.

The phytotoxicity assay provided important insights into the effects of these bioextracts on seed germination. The results suggest that both bioextracts exhibit concentration-dependent effects on germination and growth. For MBPm, low concentrations (5 mg/mL) did not significantly affect tomato seed germination, but higher concentrations (10 and 25 mg/mL) caused substantial inhibition. This pattern is consistent with the observed antifungal activity, where higher concentrations showed more pronounced effects on fungal inhibition. In contrast, MBSn promoted germination at lower concentrations (5 mg/mL), which is an interesting finding that warrants further investigation. The inhibition observed at higher concentrations (10 and 25 mg/mL) may reflect the cumulative impact of bioactive compounds on seed physiology, as well as morphological and biochemical changes. The suppression of seed germination by plant bioextracts is due to osmotic effects that slow down the water absorption process, ultimately hindering germination and specifically affecting cell elongation, which is consistent with previous studies demonstrating the dual effects of plant bioextracts on plant growth and development [28,29]. MBSn exhibited a biphasic response with enhanced germination at lower concentrations but complete inhibition at higher levels. At low concentrations, the bioactive compounds present in the bioextracts can act as signalling molecules that stimulate beneficial physiological processes, such as seed germination and growth, by improving nutrient availability, activating germination-related enzymes, and even promoting cell division. Bioextracts may help to improve seedling resilience by enhancing the antioxidative capacity of the seeds, protecting them from environmental stresses. This provides an additional benefit of using plant-based extracts in agriculture, as they can promote growth while also potentially enhancing the plants’ resistance to both biotic and abiotic stressors. However, at higher concentrations, these same compounds can reach toxic levels, interfering with fundamental processes like protein synthesis and cellular membrane integrity, leading to reduced germination and inhibited growth, a phenomenon known as phytotoxicity [30,31]. Furthermore, it has been shown that allelochemicals can enhance crop resistance to both biotic and abiotic stress [32]. These findings suggest that both the bioextracts have potential applications in seed germination regulation, with MBSn showing promise for stimulating germination at low doses, while MBPm may be more effective for inhibiting undesirable seed germination under specific agricultural conditions, particularly in the case of weeds or invasive species. While these bioextracts show promise as antifungal agents, their phytotoxic effects, particularly at elevated concentrations, may limit their use in certain agricultural contexts. Therefore, it is crucial to determine how these bioextracts interact with different plant species and the optimal concentration range that balances antifungal efficacy with minimal phytotoxicity for safe and effective use in integrated pest management (IPM) systems [14]. This requires additional field testing and fine-tuning of application rates to ensure that the bioextracts can target fungal pathogens without adversely affecting non-target crops or surrounding plant life.

The UPLC-PDA-ESI-MS/MS analysis was specifically employed to identify the major polyphenolic compounds present in both plant bioextracts. This method allowed for the detection of several known polyphenolic compounds, which are well-documented for their antimicrobial and antioxidant properties. Acteoside, identified in both MBPm and MBSn, is a phenylethanoid glycoside known for its bioactivity against various pathogens and for its anticancer, anti-inflammatory, antioxidant, and neuro-protective activities [33,34,35], which aligns with the observed antifungal properties in this study. Additionally, the chlorogenic acid and dicaffeoylquinic acid isomers identified in MBSn may contribute to its bioactivity, as these compounds have been shown to possess antimicrobial properties, particularly against fungi [4,26,33]. The identification of these compounds is consistent with other studies reporting the antimicrobial potential of *S. nigra* bioextracts, further supporting their possible use in the development of antifungal agents [4,36,37,38]. The presence of phenolic compounds such as acteoside and chlorogenic acid, known for their ability to disrupt fungal cell walls and interfere with membrane integrity, suggests that these bioextracts may inhibit fungi through membrane disruption or inhibition of ergosterol biosynthesis [25,26,38]. Such disruptions could explain the fungistatic effect observed in *C. cassiicola* and the fungicidal activity against *F. oxysporum*. The significant reduction in sporulation observed supports the hypothesis of interference with spore germination processes, a mechanism previously linked to phenolic compounds [25]. An additional feature observed during the MESn analysis was a distinct peak at 8.74 min in the EIC for *m*/*z* 353 (Panel E), as shown in Figure 9, which was not listed in Table 6 due to the absence of a confirmed identification. This peak, with an intensity comparable to that of peak 2 (9.20 min), suggests that it may correspond to an isomer of chlorogenic acid or a co-eluted compound. Further analysis would be required to confirm its identity, but the presence of this peak is noteworthy given its similarity in intensity and retention time to known compounds.

Future studies should focus on isolating and characterizing individual compounds to determine their mode of action, particularly regarding their effects on fungal cell wall synthesis, membrane integrity, and sporulation processes. Suggested approaches for further exploration include high-resolution mass spectrometry (HRMS) for precise molecular identification, nuclear magnetic resonance (NMR) spectroscopy to elucidate compound structures, and gas chromatography–mass spectrometry (GC-MS) to analyze volatile components. These advanced analytical techniques, complemented by metabolomic profiling and bioassay-guided fractionation, would enable a more comprehensive understanding of the bioextracts’ chemical composition. Such studies are crucial for uncovering the mechanisms responsible for their antifungal and phytotoxic activities, thereby enhancing their potential application in sustainable agriculture and integrated pest management strategies. Additionally, enzyme activity assays and gene expression analysis are needed to confirm the specific pathways involved. The antifungal activity of MBPm and MBSn could be further enhanced by exploring their synergistic potential with other plant bioextracts or biocontrol agents, such as *Trichoderma* spp. or Bacillus-based formulations. Synergistic effects could reduce the required concentration of each component, minimizing phytotoxicity while maximizing antifungal efficacy, which is a promising area for future research in developing combined natural fungicide formulations. Additionally, the broader applicability of these bioextracts in integrated pest management (IPM) is promising. For instance, the fungistatic and fungicidal nature of MBPm and MBSn, as observed in this work, will reduce the risk of resistance development, a persistent issue with synthetic fungicides. Their dual phytotoxic and antifungal properties suggest potential for targeted agricultural applications, such as controlling specific weed species while protecting crops from fungal pathogens. For food processing, lower concentrations of these bioextracts could be utilized as surface treatments for fresh produce, leveraging their antifungal properties to reduce spoilage without negatively affecting seed or plant growth.

## 5. Conclusions

This study reports the antifungal activity of *Plantago major* leaf bioextracts against *C. cassiicola*, *F. oxysporum*, and *P. oxalicum*, as well as *Sambucus nigra* root bioextracts. Both bioextracts demonstrated significant antifungal effects, with *P. major* exhibiting fungistatic activity and *S. nigra* showing both fungicidal and fungistatic properties. The identification of major polyphenolic compounds included acteoside, chlorogenic acid, and dicaffeoylquinic acid. Phytotoxicity assays revealed concentration-dependent effects on seed germination, with low concentrations of *S. nigra* promoting germination and higher concentrations inhibiting it. These findings suggest that these bioextracts could serve as eco-friendly alternatives to synthetic fungicides, offering sustainable solutions for integrated pest manapgement, agriculture, and the food industry. Future research should focus on isolating individual bioactive compounds and exploring their synergistic effects with biocontrol agents and expanding the profiling of bioactive compounds to include minor phenolic compounds as well as other families of bioactive compounds.

## Figures and Tables

**Table 1:** Effect of MEBPm against *P. oxalicum*. Treatments tested included the following: negative control with antifungal agent, positive control without methanolic bioextract, and concentrations of 0.16, 0.26, 0.43, 0.73, 1.21, 2, 3.4, 5.6, 8, 15, 25, and 50 mg·mL^−1^ of MEPm. C = spore development, NC = no spore development.

Treatment mg/mL	Plates	Sporulation Status	% Sporulation	Sporulation Inhibition (%)
Control (−)	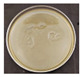	NC	0	100
Control (+)	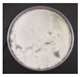	C	100	0
0.16	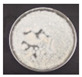	C	93.905	6.095
0.26	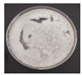	C	79.84	20.16
0.43	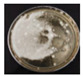	C	38.125	61.875
0.73	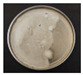	C	32.105	67.895
1.21	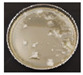	C	29.685	70.315
2	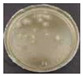	C	27.965	72.035
3.4	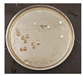	C	26.795	73.205
5.6	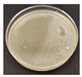	C	22.81	77.19
8	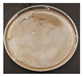	C	22.105	77.895
15	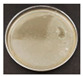	C	20.465	79.535
25	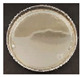	C	19.295	80.705
50	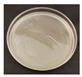	C	7.89	92.11

**Table 2:** Effect of MBPm against *C. cassiicola*. The treatments tested included a negative control with an antifungal agent, a positive control without the methanolic bioextract, and concentrations of 0.16, 0.26, 0.43, 0.73, 1.21, 2, 3.4, 5.6, 8, 15, 25, and 50 mg·mL^−1^ of MBPm. C = spore development, NC = no spore development.

Treatment (mg/mL)	Plates	Sporulation Status	% Sporulation	Sporulation Inhibition (%)
Control (−)	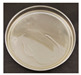	NC	0	100
Control (+)	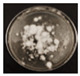	C	100	0
0.16	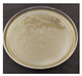	C	71.641	28.359
0.26	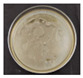	C	71.426	28.574
0.43	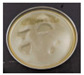	C	52.938	47.062
0.73	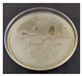	C	45.582	54.418
1.21	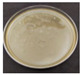	C	45.166	54.834
2	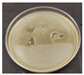	C	40.54	59.46
3.4	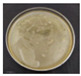	C	39.706	60.294
5.6	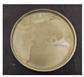	C	39.276	60.724
8	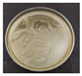	C	34.234	65.766
15	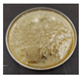	C	25.413	74.587
25	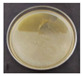	C	22.468	77.532
50	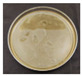	C	16.807	83.193

**Table 3:** Effect of MBSn on the percentage of sporulation against *P. oxalicum*. The treatments tested included the following: negative control with antifungal agent, positive control without methanolic bioextract, and concentrations of 0.16, 0.26, 0.43, 0.73, 1.21, 2, 3.4, 5.6, 8, 15, 25, and 50 mg·mL^−1^ of MBSn. C = spore development, NC = no spore development.

Treatment (mg/mL)	Plates	Sporulation Status	% sporulation	Sporulation inhibition (%)
Control (−)	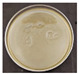	NC	0	100
Control (+)	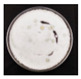	C	100	0
0.16	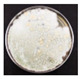	C	93.3745	6.6255
0.26	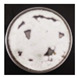	C	78.5628	21.4372
0.43	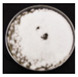	C	88.137	11.863
0.73	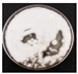	C	85.6806	14.3194
1.21	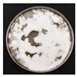	C	81.1815	18.8185
2	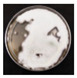	C	69.7218	30.2782
3.4	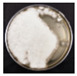	C	65.7937	34.2063
5.6	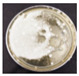	C	65.8775	34.1225
8	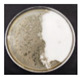	C	64.8928	35.1072
15	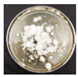	C	58.6759	41.3241
25	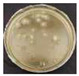	C	44.5189	55.4811
50	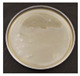	C	27.167	72.833

**Table 4:** Impact of MBPm and MBSn on final germination percentage (FG%), mean germination rate (MGR), mean germination time (MGT), and percentage inhibition (%) of *Solanum lycopersicum* seeds. All values are presented as means ± standard deviations (SDs). Different lowercase letters (a, b, c, d) indicate statistically significant differences between treatments (*p* < 0.05), determined by Tukey’s test.

Treatment mg/mL	FG %	MGR	MGT	Percentage Inhibition %
Effect of *P. major*				
5	77.77 ± 7.69	3.37 ± 0.7	3.57 ± 0.55	22.22 ± 7.69 a
10	26.66 ± 6.66	0.66 ± 0.21	4.66 ± 1.46	73.33 ± 6.66 b
25	28.88 ± 3.84	0.9 ± 0.22	6.07 ± 1.04	71.11 ± 3.84 b
Water	80 ± 11.54	2.8 ± 0.56	3.19 ± 0.26	20 ± 11.54 a
Negative control	0	0	0	100 d
Effect of *S. nigra*				
5	108.33 ± 8.33	4.46 ± 0.33	3.46 ± 0.46	0 a
10	61.11 ± 4.81	1.56 ± 0.10	4.86 ± 0.55	38.89 ± 4.81 b
25	0	0.11 ± 0.06	0	100 c
Water	80 ± 11.54	2.8 ± 0.56	3.19 ± 0.26	20 ± 11.54 d
Negative control	0	0	0	100 c

**Table 5:** UPLC-PDA-ESI-MS in the negative ion detection mode: identification of phenylethanoid glycosides in MBPm.

Peak	Rt (min) (PDA Detector)	λ max	Molecular Ion ([M–H]^−^) *m*/*z*	Fragment *m*/*z*	Putative Identification	Reference
1	10.51	328, 295sh, 224	623	461, 161, 133	Acteoside	CAS: 61276-17-3
2	11.18	327, 295sh, 218, 197	623	461, 161, 133	Isoacteoside	CAS: 61303-13-7

**Table 6:** UPLC-PDA-ESI-MS in the negative ion detection mode: identification of phenylethanoid glycosides in MBSn.

Peak	Rt (min) (PDA Detector)	λ max	Molecular Ion ([M–H]^−^) *m*/*z*	Fragment *m*/*z*	Putative Identification	Reference
1	8.74	Not identified				
2	9.17	325, 295sh, 201	353	191	Chlorogenic acid	CAS: 202650-88-2
3	10.55	328, 295sh, 224	623	461, 161, 133	Acteoside	CAS: 61276-17-3
4	11.11	327, 295sh, 218, 197	623	461, 161, 133	Isoacteoside	CAS: 61303-13-7
5	11.29	323, 295sh, 218	515	353, 179	Dicaffeoylquinic acid (isomer I)	https://massbank.eu/MassBank/RecordDisplay?id=MSBNK-RIKEN_ReSpect-PM000306&dsn=RIKEN_ReSpect (accessed on 15 October 2024)
6	11.72	327, 295sh, 218	515	353, 191, 179	Dicaffeoylquinic acid (isomer II)	https://massbank.eu/MassBank/RecordDisplay?id=MSBNK-RIKEN_ReSpect-PM000306&dsn=RIKEN_ReSpect (accessed on 15 October 2024)
7	12.42	327, 295sh, 218	515	371, 353, 191, 179, 173	Dicaffeoylquinic acid (isomer III)	https://massbank.eu/MassBank/RecordDisplay?id=MSBNK-RIKEN_ReSpect-PM000306&dsn=RIKEN_ReSpect (accessed on 15 October 2024)

## Data Availability

The original contributions presented in this study are included in the article. Further inquiries can be directed to the corresponding author.
